# Non-Invasive Ventilation: When, Where, How to Start, and How to Stop

**DOI:** 10.3390/jcm14145033

**Published:** 2025-07-16

**Authors:** Mary Zimnoch, David Eldeiry, Oluwabunmi Aruleba, Jacob Schwartz, Michael Avaricio, Oki Ishikawa, Bushra Mina, Antonio Esquinas

**Affiliations:** 1 Department of Pulmonary & Critical Care Medicine, Northwell Lenox Hill Hospital, New York, NY 10075, USA; mzimnoch@northwell.edu (M.Z.); deldeiry@northwell.edu (D.E.); oaruleba@northwell.edu (O.A.); jschwartz5@northwell.edu (J.S.); 2 Department of Pulmonary & Critical Care Medicine, Northwell Northern Westchester Hospital, New York, NY 10549, USA; mavaricio1@northwell.edu (M.A.); bmina@northwell.edu (B.M.); 3 Intensive Care Unit, Hospital Meseguer, NIV-ICM, Instituto Murciano de Investigación Biosanitaria, 30008 Murcia, Spain

**Keywords:** noninvasive ventilation, Hi Flow nasal cannula, acute hypoxic respiratory failure, hypercapnic respiratory failure, weaning from noninvasive ventilation, predictors, care settings, reintubation

## Abstract

Non-invasive ventilation (NIV) is a cornerstone in the management of acute and chronic respiratory failure, offering critical support without the risks of intubation. However, successful weaning from NIV remains a complex, high-stakes process. Poorly timed or improperly executed weaning significantly increases morbidity and mortality, yet current clinical practice often relies on subjective judgment rather than evidence-based protocols. This manuscript reviews the current landscape of NIV weaning, emphasizing structured approaches, objective monitoring, and predictors of weaning success or failure. It examines guideline-based indications, monitoring strategies, and various weaning techniques—gradual and abrupt—with evidence of their efficacy across different patient populations. Predictive tools such as the Rapid Shallow Breathing Index, Lung Ultrasound Score, Diaphragm Thickening Fraction, ROX index, and HACOR score are analyzed for their diagnostic value. Additionally, this review underscores the importance of care setting—ICU, step-down unit, or general ward—and how it influences outcomes. Finally, it highlights critical gaps in research, especially around weaning in non-ICU environments. By consolidating current evidence and identifying predictors and pitfalls, this article aims to support clinicians in making safe, timely, and patient-specific NIV weaning decisions. In the current literature, there are gaps regarding patient selection and lack of universal protocolization for initiation and de-escalation of NIV as the data has been scattered. This review aims to consolidate the relevant information to be utilized by clinicians throughout multiple levels of care in all hospital systems.

## 1. Introduction

Non-invasive ventilation (NIV) plays a central role in treating both acute and chronic respiratory failure from various causes. It offers effective respiratory support without requiring intubation, reducing the risks linked to invasive ventilation. Imperative to improving outcomes with NIV however, is selecting the right patient, using appropriate settings, recognizing early signs of deterioration, escalating care promptly, and timing the weaning process correctly. While there is widely acknowledged importance of the lattermost aspect, many weaning decisions are driven more by habit and intuition than by structured protocols, leading to inconsistent care and variable outcomes. A study by Stewart et. al. showed that before simulation-based training, healthcare providers often lacked knowledge about weaning criteria, monitoring, and when to escalate care. After implementing training and protocols, correct responses rose by 82%, highlighting the limitations of relying solely on clinical instinct [1].

Weaning from NIV is a critical step in patient recovery. When done successfully, it lowers the risk of relapses, shortens intensive care unit (ICU) and hospital stays, and improves patient comfort and outcomes. However, if weaning fails and reintubation is needed, the consequences can be severe. According to a meta-analysis of over 29,000 patients, reintubation sharply increases the risk of death, with ICU mortality rising 7.5-fold and in-hospital mortality rising 3.3-fold [2]. In liver transplant patients, mortality jumped from 5.9% to 51.2% after reintubation [3]. A major factor in this increased risk is believed to be delayed intubation due to delayed recognition of NIV failure. NIV failure is often due to subjective judgment rather than clear, evidence-based criteria and is linked to higher rates of complications and mortality [4]. Protocol-driven care, combined with proper clinician training, can significantly improve outcomes [5].

In addition to consistent protocols, research on novel markers for NIV failure is still ongoing. The COVID-19 pandemic reshaped our approach to NIV, especially in managing respiratory failure under resource constraints, and certain clinical markers—like a high neutrophil-to-lymphocyte ratio, elevated CRP, and D-dimer levels—have emerged as predictors of NIV failure and worse outcomes [6].

The COVID-19 pandemic is often seen as a stepping-stone for health care practitioners and their comfortability with NIV. Prior to the pandemic, the role of NIV remained a matter of uncertainty and discussion, and international guidelines remained inhomogeneous in their recommendations. A retrospective review of the German public health registry showed that when comparing the first and second COVID-19 waves, the first infection period had significantly more cases of immediate mechanical ventilation, without NIV as the first escalation step (75%, vs. 37% in the second wave). They showed that the second wave consequently had a higher NIV success rate (30%, vs. 9% during the first wave), and that NIV failure rates trended down between infection periods, suggesting that provider competence and comfortability continued to ameliorate between waves [7]. This article outlines evidence-based strategies for deciding when, how, and where to wean patients off NIV. The aim is to provide practical, clinician-focused guidance for making safe, individualized decisions across all inpatient locations. In 2017, the American College of Chest Physicians, European Respiratory Society (ERS), and American Thoracic Society (ATS) published guidelines on NIV for acute respiratory failure [8]. While primarily focused on initiation, these guidelines also touch on weaning, emphasizing that while we attempt to provide guidance, the approach should be tailored to each patient’s clinical status and response to treatment. The goal of the following is to provide compiled, research-based information that can be used for patients requiring NIV, from the wards to the ICU, on how and when it should be implemented and de-escalated.

## 2. Methodology

We conducted a comprehensive literature review focused on non-invasive ventilation (NIV) and the treatment of respiratory failure, critically analyzing existing evidence to assess strengths and limitations. Through this method, we were able to compile the existing literature into the following chapter to address weaning strategies from NIV. An extensive search was performed using targeted keywords—such as non-invasive ventilation, acute hypoxic respiratory failure, hypercapnic respiratory failure, and weaning from noninvasive ventilation—across PubMed, Open Evidence (OpenEvidence 2.0, Cambridge, MA, USA), and Google Scholar. A total of 58 peer-reviewed scholarly sources were identified and selected based on relevance to our research objectives. All literature discussed in the review was presented in the past tense to maintain consistency.

## 3. Epidemiology

Globally, the incidence of NIV continues to rise as national and international committees publish guidelines and recommendations endorsing its use in a myriad of pathologies [8]. In Spain, a 15-year review observed an increase in NIV use for community-acquired pneumonia (CAP) from 0.91 to 12.84 per 100,000 inhabitants [9]. Among a 15-year audit of ICUs in francophone countries, the overall use of NIV in ARF markedly increased, particularly in pre-ICU and post-extubation cohorts [10]. In terms of end-of-life care, the use of NIV has rapidly expanded over the past two decades; a nine-fold increase was observed (from 0.8% to 7.1%) over a 17-year period in a Medicare cohort study among patients hospitalized during the last 30 days of life [11]. When reviewing the use of NIV in chronic respiratory failure, data suggests underutilization of home NIV; a Canadian administrative study identified that among patients with home NIV for any indication, only 18.8% of patients had a diagnosis of COPD, despite being the most common cause of chronic hypercapnic respiratory failure [12]. Although the exact prevalence of NIV use is impossible to ascertain, health care providers are more frequently reaching for this tool amongst a wider array of clinical settings, including the emergency department, post-surgical recovery rooms, cardiology and neurology wards, and palliative care units [13].

## 4. Indications for NIV According to Recent Guidelines

When applied appropriately, NIV offers effective respiratory support, effectively avoiding endotracheal intubation and its associated risks. In primary pulmonary conditions, proper use of NIV improves gas exchange, reduces ventilation/perfusion mismatch and intrapulmonary shunting, and eases the burden on inspiratory muscles—leading to more efficient breathing. In cardiac-related respiratory failure, NIV raises intrathoracic pressure, reducing both left ventricular preload and afterload leading to improved cardiac output. Better oxygenation and ventilation often follow, which can also reverse hypercapnic encephalopathy and potentially reduce the need for sedatives [14].

Numerous guidelines outlined indications for NIV in settings of respiratory failure, including the comprehensive ERS/ATS guideline. NIV is recommended in acute respiratory failure (ARF) due to conditions such as acute exacerbation of chronic obstructive pulmonary disease (COPD), cardiogenic pulmonary edema, asthma exacerbation, obstructive sleep apnea (OSA), obesity hypoventilation syndrome (OHS), immunocompromised states, de novo ARF, viral pandemics, and chest-wall trauma [15].

In acute exacerbations of COPD, bilevel NIV is strongly recommended to prevent or treat acute or acute-on-chronic hypercapnic respiratory failure, especially in patients with a pH ≤ 7.35. A trial of bilevel NIV should be initiated in patients who are not rapidly deteriorating, to reduce the need for intubation and invasive ventilation. For patients with acute respiratory failure due to cardiogenic pulmonary edema, both bilevel NIV and CPAP are strongly recommended. These modes improve breathing effort and enhance left ventricular output through the mechanisms previously discussed [15]. 

NIV is conditionally recommended in several other scenarios, but evidence of benefits is mixed. For instance, in immunocompromised patients with mild to moderate acute respiratory failure (ARF), recent research shows no clear advantage of NIV over standard oxygen therapy. One multicenter randomized trial comparing early NIV to continuous oxygen therapy found no differences in mortality, ICU infections, duration of mechanical ventilation, or ICU length of stay [16]. A post hoc analysis of the same study also found no benefit in terms of intubation rates or survival [17]. On the other hand, in surgical patients, whom pulmonary complications are most frequently seen post-operatively after thoracic and abdominal surgeries, a randomized controlled trial (RCT) on patients following thoracic surgery showed that NIV reduced the need for intubation and lowered hospital mortality [18,19]. Another RCT compared patients with hypoxemic acute respiratory failure after abdominal surgery who received either high-flow oxygen (up to 15 L/min) or NIV. The NIV group had a significantly lower intubation rate within seven days post-operative [20]. These positive effects are thought to be from NIV’s ability to counteract atelectasis—a common complication after surgery—by recruiting atelectatic areas leading to improving lung aeration and oxygenation without causing hemodynamic instability.

NIV has also been studied in patients with non-surgical chest trauma. One RCT comparing continuous positive airway pressure (CPAP) to invasive mechanical ventilation in patients with multiple rib fractures found that those receiving CPAP had shorter ICU stays and fewer cases of pneumonia [21]. However, a later RCT in a similar patient population—with the added requirement of hypoxia—found no significant difference between the two groups [22].

We suspect that NIV is commonly clinically used in other conditions such as asthma exacerbations. However, no recommendation was made for the use of NIV in treating acute respiratory failure due to acute exacerbation of bronchial asthma, as current evidence is insufficient to support its effectiveness. Use in palliative care is also a similar situation, where recommendation to use is conditional, because if NIV is tolerated, it may help relieve breathlessness and ease the work of breathing [15]. NIV is also commonly used in the post-extubation setting, particularly for patients at high risk of extubation failure. There is data to suggest its use in this setting. For example, in a randomized controlled trial, Ferrer et al. assigned high-risk patients to either NIV or standard oxygen therapy after extubation [23]. High-risk patients were defined as those over 65 years old, those intubated due to cardiac failure, or those with an APACHE II score above 12 at extubation. The study found that NIV reduced the incidence of post-extubation respiratory failure and ICU mortality in this group [23]. Similarly, Nava et al. conducted an RCT on high-risk patients defined by criteria such as hypercapnia, heart failure, ineffective cough with excessive secretions, repeated weaning trial failures, multiple comorbidities, or upper airway obstruction [24]. Patients randomized to NIV after extubation had a lower risk of developing respiratory failure compared to those receiving standard care (Table 1) [24].

## 5. NIV Weaning: Initiation Criteria and Monitoring Guidelines

The timeline for weaning from NIV varies based on the underlying cause of respiratory failure. In all cases, weaning should begin once the patient is deemed capable of breathing independently [25]. Key indicators include clinical stability, improved gas exchange, and patient tolerance of reduced support. Furthermore, patients considered NIV weaning to show signs of improved respiratory distress. This includes reduced use of accessory muscles, a normalized respiratory rate, and visible comfort. As one may surmise, readiness for weaning is guided both clinically and objectively as outlined below [26].

Clinical Criteria:

- Effective cough and minimal secretions
- Stability of the primary issue that necessitated intubation
- Absence of acute infection

Objective Criteria:

- Hemodynamic stability: heart rate ≤ 140 bpm, systolic BP 90–160 mmHg, with minimal or no vasopressors
- Adequate oxygenation: SpO_2_ ≥ 90% on FiO_2_ ≤ 40%, or PaO_2_/FiO_2_ ≥150 mmHg
- PEEP ≤ 8 cmH_2_O
- Respiratory rate < 35 breaths/min
- Absence of significant apneic episodes
- Tidal volume ≥ 5 mL/kg
- Adequate mental status

In preparation for weaning, close monitoring is essential to identify early signs of failure. During a weaning trial from NIV, several key variables must be monitored closely to assess the likelihood of a successful outcome [27]. These include patient-related factors, ventilator settings, and physiological indicators. Vital signs, particularly respiratory rate, should be tracked for signs of hemodynamic instability. Additional signs such as increased respiratory effort, paradoxical abdominal breathing, and poor tolerance of the NIV interface can indicate excessive work of breathing, suggesting poor weaning candidacy. Level of consciousness is another critical factor, as diminished alertness may signal a persistently elevated CO_2_ level, in which NIV discontinuation can further lead to complications. On the ventilator side, clinicians should monitor tidal volumes (target 4–6 mL/kg), respiratory rate, minute ventilation, and pressure requirements. Attention should also be paid to desynchrony issues—often caused by poor respiratory effort, auto-PEEP, or excessive auto-triggering. If the ventilator alarm is triggered due to apnea, high respiratory rate, or abnormal minute ventilation, then the frequency should be trended to give further evidence of weaning tolerability. Finally, gas exchange should be evaluated through continuous pulse oximetry and arterial or venous blood gas analysis to ensure adequate oxygenation and ventilation (Table 2) [27].

## 6. Weaning Strategies and Techniques for NIV

NIV is critical tool in the physician’s approach to liberation from mechanical ventilation and the management of a myriad of respiratory diseases, but it still carries its share of complications and appropriate and timely weaning is critical [28]. Analogous to the timely extubation of a patient, NIV should be discontinued promptly after the resolution of the acute primary disorder leading to its use [29].

The challenge, however, is that unlike the weaning of mechanical ventilation, there is little data on the optimal methodology of weaning non-invasive ventilation [25,30,31]. Frequently, decisions on when, how, and where to initiate weaning from NIV stem from the expertise and daily clinical practice of clinicians. Given the spectrum of indications that NIV may assist patients and clinicians, patient-derived factors such as etiology of acute respiratory failure, disease severity, prior NIV use, and clinical status of the patient may help predict the optimal weaning method for each patient. Along with these physician-driven approaches to consider NIV weaning, discontinuation of NIV may be either patient-driven (e.g., NIV intolerance), protocol-driven, or a combination of the two. Whichever method is chosen, remaining vigilant is crucial, as premature NIV discontinuation can result in worsening respiratory status and possible reinstitution of NIV, which is particularly true in patients at risk of nocturnal hypoventilation such as severe COPD, obesity hypoventilation syndrome, and neuromuscular diseases [29].

**Figure 1:**
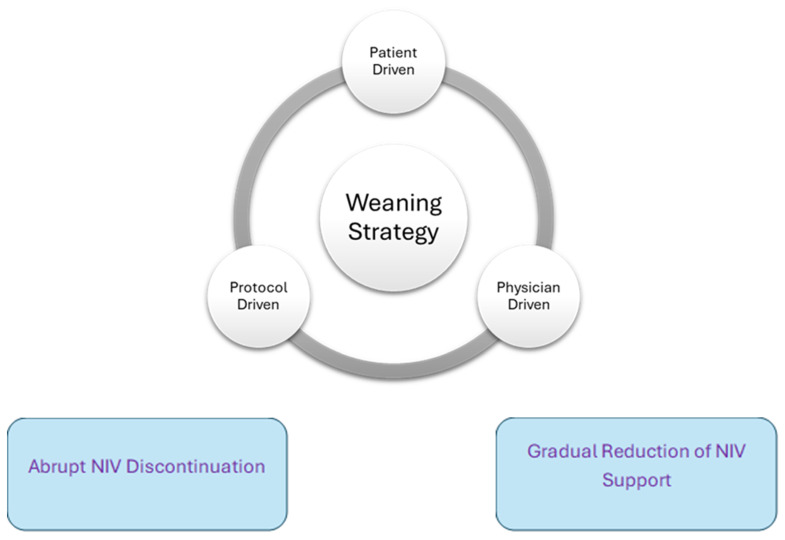
Weaning strategies and techniques.

Once a decision is made to wean, there are two strategies clinicians can employ: (i) gradual decrease in the level and/or duration of NIV support, or (ii) abrupt NIV discontinuation (Figure 1).

1. Gradual weaning

A slow weaning strategy where there is a gradual reduction of NIV intensity over several hours to days, has been associated with good clinical outcomes. This is primarily described in cases of acute hypercapnic respiratory failure, often secondary to an acute exacerbation of COPD [32].

**Figure 2:**
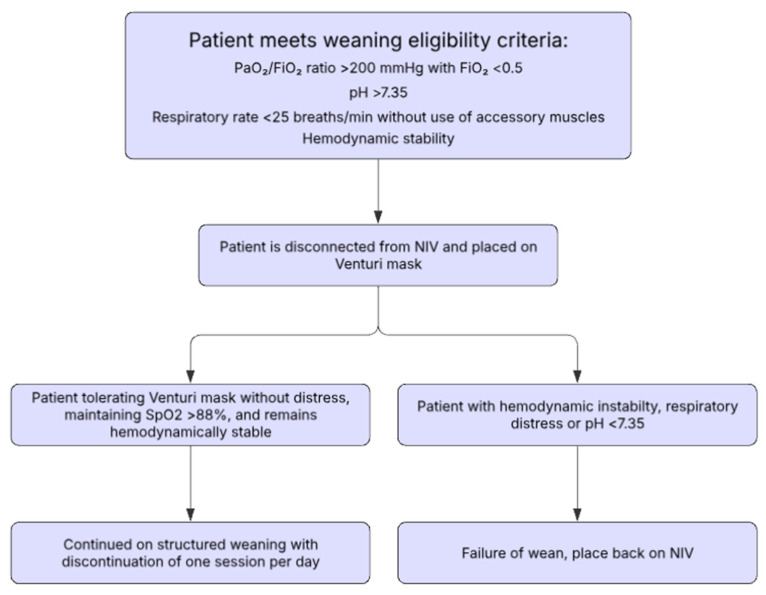
Gradual weaning algorithm as proposed by Faverio et al. [32].

One potential weaning algorithm was proposed by Faverio et al. (Table 3, Figure 2) in a retrospective study of patients suffering from an acute exacerbation of COPD with acute hypercapnic respiratory failure who received NIV [32]. Their study presented an NIV weaning protocol with gradual interruptions, pending the patient’s clinical improvement. Patients were considered for NIV weaning when they showed signs of clinical stability, including the following: PaO_2_:FiO_2_ >200 mmHg during NIV with an FiO_2_ < 0.5, a pH > 7.35 and RR < 25 without the use of respiratory accessory muscles, and hemodynamic stability. Once all these parameters were met, patients were disconnected from NIV for 1 h and were administered oxygen through a venturi mask with FiO2 being titrated to maintain a SpO_2_ of 88–92%. If the pH, RR, and hemodynamic stability were sustained, patients were placed on an NIV weaning protocol. The weaning protocol starts with three daily NIV use sessions (morning, afternoon, and evening), with each session involving continuous NIV application for at least 3 h to the maximum duration tolerated by the patient. Each session was interrupted sequentially across 3 days starting from the morning session on day 1, then morning and afternoon session on day 2, and all sessions on day 3. The treating physician determined the number of days with a pause during each NIV session before moving on to multiple pauses per day (Table 3).

The duration at each stage was determined by the treating physician. NIV settings were maintained at the individualized levels previously used to reverse respiratory failure. The number of days at each stage of weaning was at the discretion of the physician, and the NIV pressures used were maintained on the same personalized setting used to reverse their hypercapnic respiratory failure. NIV weaning failure was described as follows: pH ≤ 7.35 and/or respiratory distress, or hemodynamic instability. The primary outcomes assessing this protocol were new worsening of gas exchange following completion of the weaning protocol, and in-hospital mortality. Faverio et al. reported that patients who completed the protocol did not experience recurrence of acute hypercapnic respiratory failure or in-hospital mortality [32]. Important to identify, however, was that this study only stratified patients into a completion group (those who completed the weaning trial successfully) and a failure group (those who did not complete the weaning protocol due to intolerance, pneumothorax, or worsening respiratory failure requiring increased NIV sessions); the lack of a control group to compare different weaning methods is a critical limitation [32]. 

Other studies have shown that protocol-directed weaning, where respiratory therapists follow a structured protocol, can reduce the duration of NIV and ICU stay compared to physician-directed weaning. Particularly in COPD patients, structured weaning protocols have been associated with lower failure rates and reduced re-intubation as demonstrated by Ferrer et al. [23]. This approach has been effective in patients with chronic obstructive pulmonary disease (COPD). Duan et al. [33] compared protocol-directed by respiratory therapists versus physician-directed weaning from non-invasive ventilation (NIV); both groups had similar baseline characteristics, with most patients (64%) having COPD. Although the overall number of successful weans was similar (37 vs. 36), the protocol-directed approach significantly reduced both the duration of NIV (2.6 vs. 4.4 days, *p* < 0.001) and ICU stay (5.8 vs. 8.1 days, *p* = 0.02). In the protocol group, most weaning success occurred early, with 57% weaned by day 1 and 97% by day 3 [33].

2. Abrupt discontinuation

Abrupt discontinuation is the most used method, particularly in pediatric settings, where NIV is entirely discontinued once the patient meets the weaning criteria [34,35]. Although potentially less tolerable than gradual weaning, there are studies that conclude no difference in outcomes when compared to gradual weaning. A 2013 pilot study randomized patients with acute COPD exacerbation with acute hypercapnic respiratory failure to two study groups, comparing a stepwise versus immediate withdrawal of NIV, with a primary endpoint of NIV-withdrawal success rate. They found that these had no statistically significant difference in the success rate of NIV withdrawal [74.3% and 56% in the stepwise and immediate withdrawal groups, respectively (*p* = 0.139). However, the study was grossly underpowered to detect differences [36]. Sellares et al. [37] compared abrupt discontinuation of NIV to a fixed three-night nocturnal NIV group among patients admitted to the intermediate respiratory care units (IRCU) with a COPD exacerbation who had recovered from the acute episode. Key exclusion criteria involved previous use of domiciliary NIV. They found that patients who received nocturnal NIV for three nights after the initial recovery from an episode of acute hypercapnic respiratory failure had a significantly longer IRCU length of stay (4 (2–6) versus 5 (4–7) days, *p* = 0.036 *p* < 0.001), with no statistically significant difference in the rate of complications including relapse of acute hypercapnic respiratory failure, reintubation rate, long-term ventilator dependence, hospital stay, and 6-month hospital readmission or survival [37].

## 7. Location for Weaning

Monitoring patients with acute respiratory failure treated with non-invasive ventilation (NIV) involves a combination of clinical assessments and technological tools to ensure timely detection of complications or failure. Ergan et al. emphasize that monitoring should include regular clinical evaluations to assess the patient’s response to NIV and identify any signs of deterioration [38]. Arterial blood gas (ABG) analysis is crucial for evaluating ventilation and oxygenation adequacy. Additionally, nocturnal monitoring of transcutaneous CO_2_ (TcPCO_2_) and oxygen saturation (SpO_2_) is recommended to detect nocturnal hypoventilation and ensure effective gas exchange [38].

Advanced monitoring techniques involve utilizing data from ventilator built-in software, which provides insights into tidal volume, respiratory rate, and leak detection, aiding in assessing patient–ventilator synchrony. Ergan et al. [38] stress the importance of monitoring in settings equipped to handle potential complications, such as step-down units or ICUs, where experienced healthcare teams can promptly address issues. This comprehensive approach to monitoring is essential for optimizing NIV success and patient safety, as timely action based on monitoring variables is a key element in preventing the progression of respiratory failure. Below are the advantages and disadvantages of different types of units in a typical hospital setting.

1. ICU.

The ICU is the primary location in which acute respiratory failure may be managed with NIV. The ICU allows for the closest monitoring of patients, with the lowest nurse-to-patient ratios, typically 1:1 or 1:2. Additionally, continuous monitoring data such as continuous pulse oximetry, end tidal CO_2_, blood pressure, and electrocardiography is available, and a care team including intensivists, multiple nurses, and respiratory therapists are available in the event that the patient fails NIV. However, ICU bed availability is a major limiting factor in providing patients with close monitoring of care.

2. Step-down/high-dependency units (HDUs).

High-dependency units (HDUs) are good options outside the intensive care unit for safely managing patients requiring non-invasive ventilation in an acute setting. HDUs are specialized units that effectively act as an intermedium between the ICU and the general ward. HDUs are able to provide closer monitoring and higher levels of care compared to general medical floors. Continuous telemetry services (pulse oximetry, electrocardiography, heart rate, ventilatory alarms, and lower nurse-to-patient ratios (compared to general medical floors) are present in these units. The patients suitable for these units would be patients with increased respiratory needs who do not meet the full criteria for management in an ICU. When effectively implemented, there is data suggesting that these units can be a cost-effective implementation.

3. General medical floors.

When compared to ICUs and HDUs, nurse-to-patient levels are generally much lower, and real-time/continuous monitoring like pulse-oximetry, heart rate, and blood pressure are not regularly implemented. In these situations, the patients who will typically be indicated for these units are those who have chronic conditions more reliant on positive pressure rather than oxygenation/ventilation—such as sleep apnea. Due to the sheer number of patients in hospitals with hypoxic respiratory failure resulting in many ICUs being overwhelmed with not enough bed availability, the COVID-19 pandemic had catalyzed a shift in the utilization of NIV from being limited primarily to an ICU/stepdown setting to being seen more commonly on the floors. As more care teams working on the general medical floors have become more comfortable with NIV and have more frequently utilized NIV over time, more data has been gained to truly assess the efficacy of its use. Monti et al. conducted an international, multicenter, open-label, randomized trial to evaluate the impact of early non-invasive ventilation (NIV) in general wards for patients with mild acute respiratory failure (ARF) [39]. They found that early NIV significantly reduced the progression to severe acute respiratory failure compared to usual care. Specifically, the progression to severe ARF occurred in 18.5% of patients in the early NIV group versus 28.3% in the usual care group (relative risk 0.65, 95% confidence interval 0.48–0.90, *p* = 0.0080). However, there were no significant differences in median length of hospital stay, respiratory complications, 28-day mortality, or adverse events between the two groups. This study suggests that early NIV can be beneficial in preventing the worsening of respiratory failure in non-ICU settings and weaning can be safely performed [39].

## 8. Predictors of Successful Weaning

When assessing the success of the weaning process, there are several factors clinicians must take into consideration including respiratory parameters, level of consciousness, and imaging studies including lung ultrasound. There are several respiratory parameters that can be used to decipher a patient’s response to NIV which are summarized in Table 4.

During weaning, patients must be closely monitored for signs of respiratory distress or worsening gas exchange—key indicators of their response to non-invasive ventilation (NIV). Predictors of failure include a PaO_2_/FiO_2_ ratio ≤ 200 mmHg, respiratory rate ≥ 30 breaths/min, and tidal volume > 9 mL/kg of predicted body weight [33,40]. The PaO_2_/FiO_2_ ratio is the most widely used measure of hypoxemia. The ratio between 150 and 200 mmHg at baseline or within the first hour of NIV is strongly associated with failure, according to multivariate analyses. This highlights the importance of timing: when NIV failure occurs, it affects both its interpretation and management.

Neurological status and level of consciousnesses can also be used as predictors for successful weaning since monitoring for fluctuations aids in the assessment of early detection in respiratory fatigue. A Glascow Coma Scale score of over thirteen is generally considered indicative of a patient’s ability to tolerate weaning. Bedside lung ultrasound has also been found to play a valuable role in assessing weaning outcomes, mainly by assessing lung aeration and diaphragmatic thickening. 

The Rapid Shallow Beathing Index is a clinical tool often used to predict a patient’s readiness to be weaned from mechanical ventilation. It is calculated as the ratio of the respiratory rate over tidal volume and measured during spontaneous breathing trials. Similarly, it can be applied to non-invasive weaning trials with a threshold of less than 67.4 breaths/min/L being associated with successful weaning.

The Lung Ultrasound Score (LUS) is a quantitative tool that assigns a particular score ranging from 0 to 3, to multiple lung zones based on the presence and severity of ultrasound findings. The findings include B-lines, which are known to be indicative of increased lung density due to fluid accumulation, consolidations, and pleural abnormalities. Higher scores indicate more aeration loss and suggest an increased risk of NIV failure. More specifically, LUS scores greater than 18 have been associated with NIV failure with sensitivity ranging from 62% to 90.5% and specificity from 60% to 91.9% [41,42].

Another diagnostic scoring system with positive predictive value is the Diaphragm Thickening Fraction (DTF), which is used to evaluate a diaphragm’s ability to contract effectively with ultrasound by calculating the change in diaphragm thickness from end-expiration in end-inspiration. It can be measured at various points during therapy. Unlike the LUS, the higher the DTF value the higher the likelihood of successful weaning, whereas values less than 20% are associated with NIV failure, with sensitivity between 80% and 84.6% and specificity between 76.3% and 91.5% [41]. In a prospective study by Li et al. [42], patients who were successfully extubated had significantly higher diaphragmatic excursion (DE) and DTF compared to those who failed extubation (DE: 1.64 cm vs. 0.78 cm; DTF: 49.48% vs. 27.85%, *p* = 0.001 for both). Among individual predictors, DTF had the highest diagnostic accuracy for weaning success, with an AUC of 0.881, sensitivity of 94%, and specificity of 84%. This outperformed other measures like RSBI, LUS, and DE alone. Optimal cutoffs for predicting successful weaning were DTF ≥ 30%, DE ≥ 1.3 cm, LUS ≤ 11, and RSBI ≤ 102. Combining all four metrics (RSBI, LUS, DE, DTF) yielded the highest predictive value, with an AUC of 0.919, sensitivity of 96%, and specificity of 89%. 

Scoring systems that combine clinical signs with objective data—like the ROX index and HACOR score—can help predict non-invasive ventilation (NIV) failure and guide timely escalation. The ROX index calculates the ratio of oxygen saturation (SpO_2_) to FiO_2_, divided by respiratory rate. Though originally used for high-flow nasal cannula therapy, it is also applicable to NIV. A lower ROX index signals a higher risk of treatment failure. It is typically assessed at 2, 6, and 12 h after NIV initiation. A ROX index below 4.88 at 12 h strongly correlates with the need for intubation [43]. 

The HACOR score includes five components:

- H—Heart rate
- A—Acidosis (pH)
- C—Consciousness (Glasgow Coma Scale)
- O—Oxygenation (PaO_2_/FiO_2_)
- R—Respiratory rate

A HACOR score > 5 within the first hour of NIV is a strong predictor of failure. In one study, it had a diagnostic accuracy of 81.8% in the testing group and 86% in the validation cohort. Additionally, patients with a score > 5 had significantly higher hospital mortality (65.2%) compared to those with scores < 5 (21.6%) [44,45,46].

## 9. Predictors for Failure of Weaning and Need for Escalation

Defining failure of NIV remains a significant clinical challenge, as the term “failure” can reflect a variety of different scenarios that ultimately lead to the decision to intubate. This decision is often driven by subjective clinician judgment, or gestalt, rather than a uniform set of objective criteria. Clinicians may interpret the effectiveness of NIV differently based on their experience and perspective, especially in cases of AHRF. The absence of a universally accepted predictive score with high accuracy and validity further complicates the issue. Ultimately, the core problem lies in distinguishing between objective indicators and the true physiological mechanisms of failure versus decisions influenced by clinical subjectivity [47]. NIV failure itself can be classified by timing, however, which is associated with its underlying reasons and may guide timely escalation to invasive ventilation (Table 5) [48]. 

Interpreting the efficacy of NIV and determining whether a weaning approach is failing remains a clinical challenge. During the process of weaning, it is essential to closely monitor patients with both clinical signs and objective data. Identifying reliable parameters for NIV weaning failure is critical, as it enables timely interventions that can significantly reduce mortality and adverse outcomes. Delayed recognition of NIV failure—regardless of when it occurs—has been associated with increased risk for ICU and in-hospital mortality [8,49,50]. 

Weaning failure can manifest in various clinical scenarios, often necessitating escalation of care, such as adjusting NIV setting or proceeding with endotracheal intubation. Preventing the need for invasive mechanical ventilation should remain a priority, given its association with worsening clinical outcomes.

This task is complicated by the need for clinicians to integrate objective data with their own clinical judgment. Timely assessment and intervention are imperative, as delays in recognizing and responding to NIV failure contribute directly to patient mortality. Evidence suggests that protocolizing weaning strategies may offer a beneficial approach by standardizing care and reducing variability in clinical decision-making.

NIV failure is influenced by several factors, including the pathophysiology and severity of the underlying disease, the care team’s expertise in device application, and patient-related comorbidities such as advanced age and elevated BMI. The overall incidence of NIV failure in patients with AHRF is approximately 30%, though this rate varies based on the underlying condition. Failure is most common in patients with ARDS (51%) and community-acquired pneumonia (50%), while it is less frequent in cases of cardiogenic pulmonary edema (10%) and pulmonary contusion (18%). Multivariate analysis identified several independent predictors of NIV failure: age over 40 (OR 1.72), a SAPS II score ≥ 35 (OR 1.81), diagnosis of ARDS or CAP (OR 3.75), and a PaO_2_/FiO_2_ ratio ≤ 146 after one hour of NIV (OR 2.51). Patients who failed NIV and required intubation experienced significantly longer ICU stays, higher rates of ventilator-associated pneumonia and sepsis, and greater ICU mortality (*p* < 0.001). These findings underscore that NIV effectiveness is highly condition dependent [51]. Non-invasive ventilation (NIV) shows limited effectiveness in patients with community-acquired pneumonia (CAP), with reported failure rates between 50% and 76%. Key predictors of NIV failure include a widened alveolar–arterial gradient, persistent tachypnea, high SOFA scores, worsening radiologic findings, and sustained low PaO_2_/FiO_2_ ratios after NIV initiation. An APACHE II score greater than 15 significantly increases the risk of failure, and both APACHE II ≥ 15 and SOFA ≥ 2 are associated with higher mortality [52]. In one study by Murad et al., 76% of ICU patients with CAP failed NIV and required intubation [53]. Similarly, Carron et al. found a 56% failure rate in severe CAP cases treated with NIV [54]. These findings highlight the need for cautious use and close monitoring of NIV in CAP patients. In patients with pulmonary edema, the NIV failure rate is around 30%. Key predictors of failure include age over 75, a pre-NIV heart rate above 80 beats per minute, and urinary output less than 150 cc/hour during NIV. Severity scores such as APACHE II ≥ 15 and SOFA ≥ 2 further indicate elevated risk. Among predictive tools, a ROX score below 4.88 and a HACOR score ≥ 5 at two hours after NIV initiation are strong indicators of likely failure in this patient group [55]. In contrast, NIV shows better outcomes in neuromuscular disorders and obesity hypoventilation syndrome (OHS), where it effectively manages hypercapnic respiratory failure and decreases hospitalization. In neuromuscular diseases, respiratory muscle weakness is the central issue, leading to hypoventilation, especially during sleep, and difficulty clearing secretions—factors that increase infection risk and complicate weaning from ventilation. NIV helps by reducing respiratory muscle load and fatigue. However, NIV success varies by condition. In ALS, failure rates are higher due to disease progression and bulbar muscle involvement, which impair secretion management and airway protection. In contrast, patients with Duchenne muscular dystrophy (DMD) and spinal muscular atrophy (SMA) show higher long-term success rates with NIV—89.4% and 91.3% over five years, respectively. Myasthenia gravis patients generally respond well to NIV, particularly during myasthenic crises, where early intervention can shorten the need for ventilatory support [56,57]. Careful patient selection and close monitoring—using tools such as the ROX index and HACOR indices mentioned above, are essential to improving NIV outcomes (Table 6).

## 10. Methods of Escalation

While NIV can prevent intubation and reduce complications, its success hinges on timely reassessment and clear escalation strategies. Early recognition of NIV failure—using both clinical judgment and objective data—is critical to avoid deterioration. Frequent reassessment is essential, ideally every 1–2 h [58]. In cases of early decompensation, reverting to full ventilatory support on prior settings can buy time to identify and manage reversible issues such as fluid overload, infection, or agitation. Promptly identifying and resolving human factors such as poor mask fit, patient discomfort, and miscommunication among staff can also significantly improve outcomes. If weaning fails repeatedly, a hybrid approach may help. For example, alternating NIV with HFNC during breaks can reduce the work of breathing and aid secretion clearance [59]. Adjuncts like chest physiotherapy and devices like Aerobika (Monaghan Medical, Plattsburgh, NY, USA) may also provide benefit.

## 11. Outcomes of Failure to Wean

Failure to successfully wean from NIV often leads to worsening respiratory failure requiring intubation and mechanical ventilation. Patients who fail NIV commonly present with persistent hypoxemia and hypercapnia due to inadequate gas exchange and respiratory muscle fatigue [60]. A 2016 retrospective study found that 30.8% of patients failed NIV required intubation, which was associated with ICU stays averaging 4 days longer, lower survival rates (OR 0.10 [0.02–0.59]), and increased risk of mortality compared to those successfully weaned [60].

NIV failure also significantly increases cardiovascular stress. Heightened respiratory effort and poor gas exchange raise oxygen demand, triggering events such as arrhythmia or myocardial ischemia, particularly in patients with underlying heart conditions [35]. The transition from positive-pressure ventilation to spontaneous breathing can also cause hemodynamic shifts that may have deleterious effects including elevated left ventricular filling pressure, induction of weaning-induced pulmonary edema (WIPO), and exacerbating pre-existing cardiac conditions [61]. This is likely the reason why clinical indicators like elevated heart and respiratory rates predict NIV failure illustrating cardiovascular strain [62]. In terms of these hemodynamic effects on clinical outcomes of cardiac patients, a study by Metkus et al. found a 26.5% 30-day mortality in patients with acute heart failure who failed NIV vs. 5.6% in those who succeeded [63]. The same study also found that NIV failure nearly doubled in-hospital mortality risk (OR 1.95 [95% CI: 1.59–2.40]) [63].

NIV failure also leads to significantly longer ICU and hospital stays. In one comparative cohort study by Meeder et al., NIV failure increased ICU stay by an average of 4 days (OR 1.16 [1.04–1.30]), with an overall association to lower survival rates [60]. A separate study by Correa et al. reported a median ICU stay of 12 days in NIV failure vs. 2 days in successful weaning, with a median hospital stay of 30 days vs. 15 days [50].

Failure to wean off NIV is also associated with increased mortality, particularly in patients with acute respiratory failure. A study published by Burns, et al. found that patients who failed NIV had significantly lower survival rates compared to those who successfully weaned, with an odds ratio of 0.10 for survival in the NIV failure group [59]. This highlights the critical impact of NIV failure on patient outcomes, emphasizing the need for timely intervention and appropriate patient selection. 

Furthermore, a systematic review and meta-analysis by Burns, et al. demonstrated that non-invasive weaning significantly reduced mortality compared to invasive weaning, a risk ratio of 0.57 [59]. This suggests that successful weaning from NIV can substantially improve survival rates. This has also been corroborated in the findings of Munshi, et al., indicating that early liberation from invasive ventilation through NIV can reduce mortality, particularly in patients with chronic obstructive pulmonary disease (COPD) [35]. These studies collectively underscore the importance of effective NIV weaning strategies to mitigate the risk of increased mortality.

NIV wean failure can also lead to increased rates of nosocomial infections, such as ventilator-associated pneumonia. Prolonged use of NIV, especially when it fails to adequately support respiratory function, can increase the risk of nosocomial pneumonia. A study by Zhang, et al. found that nosocomial pneumonia occurred in 3.1% of patients on NIV, with a higher incidence in those requiring longer durations of NIV support [64]. This increased duration of NIV use can lead to higher exposure to potential pathogens, thereby elevating the risk of infection [64].

The risk of VAP is notably lower with NIV compared to invasive mechanical ventilation, as highlighted by Burns, et al., which found that non-invasive weaning strategies significantly reduced the incidence of VAP (risk ratio 0.25) compared to invasive weaning [59]. However, when NIV fails and intubation becomes necessary, the risk of VAP increases due to the invasive nature of endotracheal intubation and the associated prolonged mechanical ventilation. This transition can lead to a higher incidence of VAP, as invasive ventilation is a well-known risk factor for this complication.

Moreover, the failure to wean off NIV and the subsequent need for intubation can lead to longer ICU stays, which further increases the risk of nosocomial infections. The increased length of stay provides more opportunities for exposure to hospital-acquired pathogens, compounding the risk of developing infections such as VAP. Therefore, timely and successful weaning from NIV is crucial to minimizing these risks and improving patient outcomes.

Neurological issues commonly seen in the inpatient setting, including agitation, delirium, and cognitive impairment may also worsen with NIV failure. Contributing factors include increased work of breathing, persistent or worsening abnormal gas exchange with hypoxia and hypercapnia, and need for sedation. These neurocognitive complications can delay recovery and complicate care, emphasizing the need for timely and effective weaning strategies. 

## 12. Limitations

There are several important limitations to recognize when considering non-invasive ventilation. We discussed a portion of these as they relate to the success of this therapy and predicators of failure. Contraindications such as hemodynamic instability, altered mentation, excessive secretions and ill-fitting masks all compromise the efficacy of NIV therapy. Efficacy is also highly dependent on the underlying etiology and severity of a patient’s respiratory failure. In cases with higher failure rates, prolonged NIV trials and delayed escalation to mechanical ventilation becomes its own risk factor for increased patient mortality.

There are also significant gaps in the evidence used to support NIV that constrain its broader application; they range from patient selection, practice/implementation heterogeneity, and lack of protocolization. Regarding patient selection, although it is widely accepted that the use of NIV in older adults and immunocompromised patients may reduce intubation rates, there is marginal evidence of its mortality benefit in these high-risk populations. We have discussed the subjective nature of practice as it pertains to weaning and escalation decisions. While many of the choices are made by incorporating objective measures such as PaO_2_/FiO_2_, Rapid Shallow Breathing Index, ROX index, LUS, and DTF scores, the ultimate decision relies on clinician judgment. While local or hospital-system based protocols may exist, there is not a universally accepted, protocolized criteria. Heterogeneity can be viewed as a necessary benefit given the complexity and diversity of patient cases; however, the lack of standardization leads to high variability in practice and limited generalizability in widespread application. The care setting in which much of the evidence supports non-invasive ventilation should also be considered. Most research has been obtained from ICU settings with specialized staff and resources only found in high-acuity settings. Some of the objective measures are often operator dependent and can be easily influenced by patient positioning, timing, and frequency of assessment, etc. It begs the question: how reproducible is the current evidence in step-down units and general wards when resources may be limited and monitoring capacity is reduced?

## 13. Future Directions

Further research in the strategies and approaches to weaning will allow for improved outcomes; while there have been pilot studies comparing a gradual and a more aggressive approach, stratifying and defining both gradual and abrupt weaning approaches beyond two broad terms may also allow for a more flexible and patient-centered approach.

There is still a need for an evidence-based, tangible, and reliable set of criteria or a scoring tool specifically used in NIV. There are many different objective scoring systems which do provide some predictive value in the success and failure in NIV. Additionally, while there is an abundance of literature regarding the ideal criteria for weaning off invasive ventilation, the current literature available on weaning criteria for NIV and the outcomes associated with these criteria are still lacking. Further exploring the rates of successful weaning when using an objective, identifiable set of criteria would allow practitioners to utilize a more concrete and evidence-backed tool rather than relying on individual and anecdotal judgment.

As mentioned earlier, while the ICU is the primary and objectively most ideal place for weaning off NIV, further research focused on providing appropriate protocols for NIV weaning on HDUs and general medical floors would improve the overall cost-effectiveness in healthcare systems.

## 14. Conclusions

Effective weaning from NIV is a critical determinant of patient outcomes in acute and chronic respiratory failure. Despite its widespread use, current practice often lacks standardized protocols, relying instead on clinician judgment that can lead to delays in escalation, increased morbidity, and mortality. Evidence clearly supports the use of structured, protocol-driven weaning strategies—tailored to patient-specific factors—to reduce the risk of failure, shorten ICU and hospital stays, and improve survival. Predictive tools like the ROX index, HACOR score, and diaphragm ultrasound metrics (DTF, LUS) offer valuable guidance but require broader integration into routine practice. Additionally, location of care plays a vital role, and emerging data supports the safe implementation of NIV and its weaning even in non-ICU settings, provided proper monitoring is in place. Despite the evidence that has been presented, it is important to acknowledge the limitations in patient selection, practice/implementation heterogeneity, and lack of protocolization. Future research must focus on optimizing weaning protocols across varied care environments, particularly step-down units, and refining objective criteria to predict failure early. Ultimately, timely, individualized, and data-informed weaning from NIV is essential for maximizing its life-saving potential and minimizing harm.

## Figures and Tables

**Table 1:** The abridged recommendations of NIV use adapted from the ATS/ERS guidelines.

Indication	Recommendation for Use of NIV Based on ERS/ATS Guidelines	Strength of Recommendation
Moderate-to-severe COPD causing respiratory acidosis	Bilevel NIV for patients with ARF leading to acute or acute-on-chronic respiratory acidosis (pH ≤ 7.35) due to COPD exacerbation	Strong recommendation, high certainty of evidence
	Trial of bilevel NIV in patients considered to require endotracheal intubation and mechanical ventilation, unless the patient is immediately deteriorating	Strong recommendation, moderate certainty of evidence Strongest recommendation is in patients with pH 7.25–7.35
ARF from acute cardiogenic pulmonary edema	Either bilevel NIV or CPAP for patients with ARF due to cardiogenic pulmonary edema	Strong recommendation, moderate certainty of evidence
ARF in immunocompromised patients	Early NIV for immunocompromised patients with ARF	Conditional recommendation, moderate certainty of evidence
Post-operative ARF	Use of NIV for patients with post-operative ARF	Conditional recommendation, moderate certainty of evidence
ARF from chest trauma	Use of NIV for chest trauma patients with ARF	Conditional recommendation, moderate certainty of evidence
ARF in palliative care	Offering NIV to dyspneic patients for palliation in the setting of terminal cancer or other terminal conditions	Conditional recommendation, moderate certainty of evidence
Aid in weaning from mechanical ventilation in hypercapnic patients	NIV can be used to facilitate weaning from mechanical ventilation in patients with hypercapnic respiratory failure	Conditional recommendation, moderate certainty of evidence
Prevention post-extubation respiratory failure	Non-high-risk patients: NIV should not be used to prevent post-extubation respiratory failure	Conditional recommendation, very low certainty of evidence
	High-risk patients: NIV should not be used to prevent post-extubation respiratory failure	Conditional recommendation, low certainty of evidence
Treatment of post-extubation respiratory failure	NIV should not be used in the treatment of patients with established post-extubation respiratory failure	Conditional recommendation, low certainty of evidence
ARF in acute asthma	Unable to offer a recommendation	
ARF in pandemic viral illness	Unable to offer a recommendation	
Aid in weaning from mechanical ventilation in hypoxic patients	Unable to offer a recommendation	

**Table 2:** Key factors to be monitored during weaning trials.

Patient factors: ο Respiratory effort ο Use of accessory muscles or paradoxical breathing ο Comfort with the NIV interface ο Level of consciousness—decreased alertness may signal rising CO_2_ and raises aspiration risk
Physiological parameters: ο Respiratory rate and vital signs to catch hemodynamic instability ο Tidal volume (target 4–6 mL/kg) ο Minute ventilation ο Air leak volume (<25 L/min) Ventilator indicators: ο Desynchrony from poor effort, auto-PEEP, or inappropriate trigger settings ο Alarm trends: apnea, high respiratory rate, or abnormal minute ventilation
Gas exchange: ο Continuous pulse oximetry ο Arterial or venous blood gas monitoring

**Table 3:** Gradual weaning algorithm, as proposed by Faverio et al. [32].

**Weaning Eligibility Criteria:** Patients were considered for weaning when the following criteria were met during NIV: PaO_2_/FiO_2_ ratio > 200 mmHg with FiO_2_ < 0.5 pH > 7.35 Respiratory rate < 25 breaths/min without use of accessory muscles Hemodynamic stability
**Initial Assessment:** Once criteria were met: Patients were disconnected from NIV for 1 h and transitioned to oxygen via a Venturi mask FiO_2_ was titrated to maintain SpO_2_ between 88 and 92% If respiratory and hemodynamic stability persisted, the patient proceeded to a structured weaning protocol
**Weaning Protocol:** NIV was administered in three daily sessions: morning, afternoon, and evening Each session lasted a minimum of 3 h, or longer if tolerated Weaning proceeded by gradually discontinuing one session per day: **Day 1**: Interrupt morning session **Day 2**: Interrupt morning and afternoon sessions **Day 3**: Interrupt all sessions The duration at each stage was determined by the treating physician. NIV settings were maintained at the individualized levels previously used to reverse respiratory failure.
**Weaning Failure Criteria Were Defined As:** pH ≤ 7.35 Signs of respiratory distress Hemodynamic instability

**Table 4:** Parameters of successful weaning from NIV.

PaO_2_/FiO_2_ ratio greater than 200 at one hour after NIV initiation
Respiratory rate < 20–22 breath/min without signs of distress
Tidal volumes less than 9 mL/kg of predicted body weight at one hour after initiation of NIV
pH > 7.33
PaCO_2_ normalization to baseline
Glascow Coma Scale score of over 13
Rapid Shallow Beathing Index (RSBI) threshold of less than 67.4 breaths/min/L
LUS scores less than 18
Higher diaphragm thickness fraction (DTF)
ROX Index > 4.88 after 2 h
HACOR Score < 5

**Table 5:** Timing of NIV failure and associated causes.

Immediate failure (within minutes to <1 h):	Often due to secretion retention, hypercapnic encephalopathy, patient intolerance, agitation, or NIV asynchrony.
Early failure (1–48 h):	Typically caused by persistent gas exchange abnormalities, worsening acute illness, or unrelieved respiratory distress.
Late failure (>48 h)	Occurs after an initial improvement, often linked to sleep disruption and serious comorbidities.

**Table 6:** Factors associated with weaning failure from non-invasive ventilation (NIV).

Category	Risk Factor
**Respiratory Parameters**	PaO_2_/FiO_2_ ratio ≤ 200 mmHg PaO_2_/FiO_2_ ratio between 150 and 200 mmHg (baseline or within 1 h of NIV) Higher PaCO_2_, lower pH, after one hour of NIV initiation Respiratory rate ≥ 30 breaths/min Tidal volume > 9 mL/kg predicted body weight ROX Index < 4.88 at 12 h HACOR Score > 5 within 1 h of NIV
**Timing of Failure**	Failure within 1 h of NIV initiation (“early failure”)
**Technical Issues**	Poor mask fit Mask air leaks Excessive secretions Inadequate humidification
**Patient Tolerance**	Agitation Anxiety Discomfort or intolerance to the mask
**Underlying Conditions**	Persistent hypoxemia Worsening hypercapnia Unresolved primary illness
**Hemodynamic Instability**	Need for vasopressors/inotropes Arrhythmias
